# Ability self-concept and self-efficacy in higher education: An empirical differentiation based on their factorial structure

**DOI:** 10.1371/journal.pone.0234604

**Published:** 2020-07-21

**Authors:** Henrike Peiffer, Thomas Ellwart, Franzis Preckel

**Affiliations:** Department of Psychology, University of Trier, Trier, Germany; Murcia University, Spain, SPAIN

## Abstract

Ability self-concept (SC) and self-efficacy (SE) are central competence-related self-perceptions that affect students’ success in educational settings. Both constructs show conceptual differences but their empirical differentiation in higher education has not been sufficiently demonstrated. In the present study, we investigated the empirical differentiation of SC and SE in higher education with *N* = 1,243 German psychology students (81% female; age *M* = 23.62 years), taking into account central methodological requirements that, in part, have been neglected in prior studies. SC and SE were assessed at the same level of specificity, only cognitive SC items were used, and multiple academic domains were considered. We modeled the structure of SC and SE taking into account a multidimensional and/or hierarchical structure and investigated the empirical differentiation of both constructs on different levels of generality (i.e., domain-specific and domain-general). Results supported the empirical differentiation of SC and SE with medium-sized positive latent correlations (range *r* = .57 - .68) between SC and SE on different levels of generality. The knowledge about the internal structure of students’ SC and SE and the differentiation of both constructs can help us to develop construct-specific and domain-specific intervention strategies. Future empirical comparisons of the predictive power of SC and SE can provide further evidence that both represent empirical different constructs.

## Introduction

Students’ self-perceptions are core constructs in educational research [1], and numerous studies have investigated the constructs of ability self-concepts and self-efficacy [2]. Ability self-concepts (SC) comprise mental representations of students’ own abilities in academics in general or in specific academic domains [3]. Self-efficacy (SE) refers to students’ convictions that they can master given academic tasks at designated levels [4, 5]. SC and SE have much in common, for example, their emphasis on perceived competences [6]. However, they differ regarding their composition: While SC comprises affective and cognitive components, SE is conceptualized by cognitive components only. Further, both constructs differ regarding their preferred measurement strategy: While SC is assessed as general and domain-specific, SE is usually assessed as task-specific [7,8]. Moreover, SC is strongly influenced by frames of reference against which students judge their own abilities, but SE is foremost formed through prior mastery experiences [2, 9]. Despite these similarities and differences, there are only few studies investigating whether SC and SE represent two empirically distinct constructs. This lack of empirical differentiation can lead to *jingle-jangle fallacies* [10] in which researchers use dissimilar names for scales that actually assess the same construct [6]. In the present study, we therefore investigated the empirical differentiation of SC and SE.

Most studies that have investigated empirical differences between SC and SE were conducted in K-12 educational settings (e.g., [11, 12]). To our knowledge, the empirical differentiation of SC and SE has only been investigated in seven studies with higher education samples. However, the focus in these studies was limited to specific academic tasks (e.g., mathematical problem solving, [13]), or not directly related to the empirical differentiation of SC and SE but rather to the predictive power of SC and SE [e.g., 14], or of SC, SE, and further constructs such as satisfaction [15] for academic outcomes. Hence, in our study we focus on the empirical differentiation of SC and SE in higher education analyzing the internal structure of SC and SE first. This approach has been realized in K-12 educational settings [e.g., 12] but only once in higher education [16]. In addition, studies are needed that investigate relations of SC and SE in different academic domains or disciplines in higher education, because both constructs might vary over domain [17, 18]. Thus, research in different domains and disciplines is needed to learn about the generalizability of findings. In our study, we investigated the empirical differentiation between SC and SE in a large sample of psychology students.

There are some similarities between the K-12 educational setting and the context of higher education. For example, in both contexts, the transfer of knowledge and the possibility to experience academic successes and disappointments alter SC and SE [19]. Nevertheless, there are substantial differences between K-12 school systems and higher education. University students are provided with advanced knowledge in particular study-related domains, whereas students in the K-12 educational setting are equipped with wide skills and knowledge useful in broad areas of later life [19]. Because of these and further differences between both educational settings, analysis of the empirical differentiation of SC and SE in higher education extends existing knowledge of students’ competence-related self-perceptions to other student samples.

Finally, when investigating the relation of SC and SE, there are some critical methodological issues that should be considered [20]. First, previous studies have assessed SC and SE at different levels of specificity; that is, SC is typically assessed at a more general level than SE. However, SC and SE become increasingly similar when measured at the same level of specificity [14, 21]. Second, educational research has primarily compared SC and SE within only a single academic domain (e.g., mathematics; [13]). Thus, results of empirical differences are limited to that specific domain, and they cannot be generalized to further academic domains without further testing. More importantly, considering only a single academic domain prevents the comparison of both constructs with regard to their domain-general or domain-specific factorial structure. However, matching levels of specificity (i.e., domain-specific or domain-general) is a precondition for valid construct comparisons [14, 22]. Third, a number of studies comparing SC to SE measured SC using both cognitive- and affect-related items but measured SE solely through cognitive SE items (e.g., [23]). Investigating whether SC and SE are empirically distinct constructs might therefore be confounded by the inclusion of affective components within the SC measure [20].

In the present study, we controlled these methodological shortcomings of prior research by assessing SC and SE at the same levels of specificity using only cognitive items. Measuring both constructs at the same levels of specificity requires an understanding of their factorial structure. Therefore, we initially analyzed the factorial structure of university students' SC and SE while considering SC and SE in multiple academic domains. Based on the findings of their factorial structure, we examined the empirical differentiation between SC and SE at different and matched levels of specificity and in different academic domains.

To conclude, this study contributes to the generalizability of findings from K-12 settings regarding the empirical differentiation of SC and SE to university students in studying psychology, taking into account central methodological requirements that, in part, have been neglected in prior studies. Findings from this line of research can support researchers when designing construct-specific interventions for fostering SC or SE [24] in order to support university students’ academic and professional success [25, 26].

## Ability self-concept and self-efficacy: Conceptual and operational similarities and differences

In educational settings, SC comprises ability-related evaluative self-perceptions [27] that refer to how individuals view their abilities in general or in specific academic domains (e.g., [1]). Concerning the formation of students’ SC, the most relevant principles are processes and feedback of comparisons. Students compare their own skills with the perceived skills of other students (e.g., classmates) who are within their reference group (i.e., social comparisons within an external frame of reference; [28, 29]). Students further compare their own abilities across different academic domains like mathematics and English (i.e., dimensional comparisons within an internal frame of reference; [30]). Additionally, the causes to which students attribute previous success and failure influence subsequent SC (causal attributions; e.g., [31]). This SC, in turn, affect later attributions (e.g., [32]). Reflected appraisals from significant others [2] and prior mastery experiences are further environmental influences that form students’ SC [33, 34]. When measuring SC, students report their general appraisal of doing well or poorly in a given academic domain or in academics in general [2]. SC measures often ask for affective as well as cognitive self-perceptions of one’s own competence [35]. In particular, items assessing students’ SC typically ask the individual to judge his or her past performances (e.g., “I have always done well in …”; [23]).

Compared to the large body of research on SC in educational contexts, research on SE is relatively scarce [2]. Bandura [36] introduced the concept of SE in his Social Cognitive Theory (SCT), defining it as “beliefs in one’s capabilities to organize and execute the courses of action required to produce given levels of attainments” ([37], p. 16). SE beliefs result particularly from past experiences [2, 36]. These mastery experiences are specifically relevant for the development of SE: Experiences interpreted as successful raise one’s SE; experiences interpreted as unsuccessful lower it [38, 39]. Additionally, students form their SE by observing others performing a task and subsequently evaluating their own probability of success at the same task (vicarious experiences; [39]). Verbal persuasion and feedback (e.g., about one’s writing skills; [40]) from significant others (e.g., teachers) also influence one’s SE [2]. When measuring SE, it is important to specify the task that is being addressed, because SE is a cognitive perception of one’s own capacity to perform well on a specific task [36, 41, 42]. Furthermore, SE is sensitive to contextual variation in a particular task [8]. Hence, items assessing SE in academic settings generally address a specific academic task within a specific domain [21].

## Empirical differentiation of ability self-concept and self-efficacy

Despite the widely accepted conceptual and operational differences between SC and SE [2, 7, 43], there is evidence that both self-perceptions are equally good predictors for academic achievement in educational settings [e.g., 8, 44, 45]. Moreover, academic outcomes like achievement motivation or academic emotions are positively related to students’ SC and SE [39, 46–48]. For example, SC and SE show positive correlations with achievement of medium size in school and university settings [26, 49]. Due to their comparable predictive power for academic outcomes, researchers have questioned if the two constructs differ empirically. The majority of studies have investigated the empirical differentiation between SC and SE in the K-12 educational setting using structural equation modeling. For example, Scherer [12] investigated 459 German high-school students in grade levels 10 to 13, and he found evidence for distinct, but correlated SC and SE factors (latent correlation of *ρ* = .78) within the domain of chemistry. Likewise, Lee [50] showed that SC and SE form two distinct latent factors in the domain of mathematics in a sample of 250,000 15-year old students in 41 countries. Based on a secondary analysis of the Belgian data from the 2003 PISA survey, Ferla et al. [11] also found evidence for an empirical distinction between SC and SE in the domain of mathematics with a positive relation between both constructs (*r* = .37; *p* < .01). Marsh et al. [9] studied 3,350 German students in the domain of math and found medium-sized positive latent correlations between SC and SE in grades 5 to 8 (.27 in grade 5, .63 in grade 7, and .58 in grade 8). To our knowledge, there are only seven studies that assessed SC and SE in higher education. With regard to the empirical differentiation of both constructs, some studies investigated the factorial structure of the constructs and others tested and compared their criterion-related validity for explaining academic outcomes. Peterson and Whiteman [51] investigated the relation between SC and SE in higher education within a structural equation model of various self-beliefs for university students in different countries (e.g., New Zealand) and courses of study (e.g., education). As in K-12 educational settings, the authors found evidence for an overlap between SC and SE factors (*r* = .43) indicating that SC and SE are positively correlated but distinct constructs in higher education. Lent et al. [16] assessed academic self-concept, global academic self-efficacy, and domain-specific mathematics self-efficacy in 205 students from introductory psychology courses at a large midwestern university; they found that a model with separated factors for each of the variables best explained the data. Choi [14] investigated the predictive power of SC and SE in a higher education sample of 230 undergraduate students enrolled in four general education classes at a southeastern university. He found both constructs to be highly correlated (.81), but SE to be a better predictor for academic achievement than SC. Finally, Mills, Pajares, and Herron [52] assessed 303 college students enrolled in French courses at three institutions of higher education. The authors conducted hierarchical multiple regression analyses to investigate the predictive power of SE and SC (and further variables like French learning anxiety) and found SE for self-regulated learning to be the best predictor of achievement.

Of note, all studies (with the exception of Lent et al. [16]) investigating the empirical differentiation of SC and SE in diverse educational settings did not first test for their factorial structure. However, this test is crucial prerequisite to investigate the empirical differentiation of SC and SE on different and matched levels of specificity.

## The structure of self-concept and self-efficacy

A prominent self-concept model used in educational psychology was introduced in the 1970s by Shavelson and colleagues [53]. In this model, self-concept is multidimensional and hierarchically structured with a general self-concept at the top of the self-concept hierarchy. At the next level, the authors postulated a general academic SC and three non-academic components, and they further differentiated general academic SC into various domains according to different school subjects (e.g., mathematics). In ensuing years, Marsh and colleagues analyzed the Shavelson model in several studies [54, 55] and postulated the Marsh/Shavelson model, distinguishing between the two (nearly) uncorrelated second-order SC factors of mathematics SC and verbal SC. However, there was a lack of evidence supporting the existence of mathematics and verbal higher-order SC factors when considering a broader scope of academic domains [56, 57]. In addition, there were unexpected correlations between general and domain-specific SC [54]. Therefore, Brunner et al. [3] developed the nested Marsh/Shavelson (NMS) model. In this model, a single general factor forms the apex of the academic SC hierarchy, which is in accordance with the original model of Shavelson and colleagues [53]. This general factor represents the common variance of all items assessing domain-specific and general SC, whereas the specific variance explained by each SC domain is separated from the variance explained by the general factor. Today, in K-12 educational settings, there is ample evidence for the multidimensionality of SC with a general SC factor at the apex [58, 59].

In higher education, to our knowledge, there are only few studies in education that include an analysis on the factorial structure of SC. Paulick et al. [60] investigated the relations between SC and corresponding achievement in different domains of 430 pre-service biology teachers and found evidence for a multidimensional structure of SC. Yeung et al. [61] examined SC of students in a school of commerce in Hong Kong (*N* = 212) and found a multidimensional structure of SC with the domains of English, Chinese, math and statistics, economics, and principles of accounting, as well as a general SC. Further, Lau et al. [62] examined the multidimensional and hierarchical nature of English SC in 321 university students and found positive relations between a higher-order English SC and SCs of different English skills (e.g., speaking). Finally, Yeung et al. [63] studied 298 teacher education students and found that SCs in four art areas were explained by a higher-order creative arts SC factor, supporting hierarchical relations of the domain-specific SCs. The authors further studied samples of English students and students in a school of commerce; for both samples, there was evidence for hierarchical relations (i.e., global SC factor) of the domain-specific SCs.

Relative to SC, there has been little research on the structural characteristics of SE in academic settings. Some authors suggest that students form differentiated perceptions of their own capability across diverse tasks within a domain [5, 64]. Others assume that SE is linked to distinct realms of functioning, and students differentiate between different academic domains in their SE judgments [65, 66]. Furthermore, there is a debate on the presence of a general SE factor analogous to general SC [58]. Some authors propose that SE might be less general and only depend on specific tasks and contexts [8, 41], while others expect a higher-order SE factor that generalizes over several domains [67]. In this regard, Bong [4] was one of the few researchers who found empirical evidence for higher-order SE factors (mathematics and verbal) that accounted for common variance of different first-order factors of six academic subjects (algebra, geometry, U.S. history, chemistry, English, and Spanish) in a sample of high school students. In a recent study with a large sample of German students in grades 5 to 8 in the domain of math, Marsh et al. [9] found no evidence for a higher-order SE factor; rather, a generalized SE factor formed one higher-order factor with SC, and it was functionally separable from task-specific SE (e.g., with regard to predictive validities and frame-of-reference effects). However, this study considered only one domain (i.e., math). Overall, these results indicate that, in K-12 educational settings, SE might be multidimensional with respect to different academic domains, but there is no clear evidence that SE is also hierarchically organized with a general component at the apex of the hierarchy. Concluding, findings of the internal structure of SE for K-12 educational settings are inconsistent. In higher education, however, there is a lack of research on the structure of SE but also of SC as a whole [e.g., 13, 14], although knowledge of their factorial structure is needed for an empirical differentiation of SC and SE [20].

## The present study

Studies investigating the empirical differentiation of SC and SE in higher education are scarce (e.g., [13, 14, 51]) as most studies focused on K-12 settings [e.g., 11, 12]. Therefore, the main aim of the present study was to investigate the empirical differentiation of SC and SE in higher education. By doing so, we contribute to our knowledge on the generalizability of findings from K-12 settings to higher education.

We focused on studies of psychology in higher education, investigated a large student sample, and accounted for critical methodological issues that have been neglected in previous studies. That is, we assessed SC and SE in multiple domains (in the applied domains of industrial and organizational psychology, clinical psychology, and educational psychology as well as for psychology in general) using only cognitive items. To examine the empirical differentiation at different and matched levels of specificity, we initially investigated the factorial structure of SC and SE, comparing different structural models. These structural models postulate a multidimensional and/or a hierarchical structure of SC and SE as found primarily in K-12 educational settings [2–4].

Based on the findings for the factorial structure of SC and SE, we investigated the empirical differentiation of SC and SE. Using confirmatory factor analysis and structural equation modeling, we examined the empirical differentiation of SC and SE factors at the domain-specific level (e.g., educational psychology) and at the general level (e.g., psychology in general). More specifically, we tested, at different levels of specificity, whether common SE/SC factors better represented the data than separate factors for SE and SC. In line with previous findings in K-12 educational settings [e.g., 12], we hypothesized that both constructs form empirically distinct but positively correlated factors within each psychological domain and at each level of specificity.

## Method

### Participants and procedure

In the spring semester of 2015 and the fall semesters of 2015 and 2016, *N* = 1,243 undergraduate and graduate psychology students (80.8% female) from a southwestern German university completed a questionnaire during lectures at the beginning of the semester, either as a web (*n* = 321) or as a paper-and-pencil questionnaire (*n* = 901). For 21 students, the information on type of questionnaire (i.e., web-based vs. paper-and-pencil) was missing. Students' mean age was *M* = 23.62 (*SD* = 3.53) years; *n* = 428 students had already finished their undergraduate psychology studies, *n* = 813 students were enrolled in graduate school, and two students did not provide this information. Survey design, aspects of data anonymization, and ethical guidelines (based on the requirements of the American Psychological Association, 2002) were presented and approved at the 5th Department Conference Psychology, Trier University on November 21, 2012. The university ethics board confirmed that the present study is exempt from the requirement for ethical review because the research presented no risk to the participants. The verbal consent procedure was approved because the study involved anonymous questionnaires. Prior to their participation, students were informed about the following: (1) the purpose of the study, the procedures, and study duration; (2) that participation is voluntarily and that it may be terminated at any point; (3) that there are no potential risks, discomfort, or adverse effects with regard to participation; (4) that data is collected anonymously, so that no written consent would be obtained, and the verbal consent would not explicitly be asked for in order to guarantee anonymity in the context of group testing. The respondents were further informed that they expressed their consent by completing the questionnaire and their rejection by nonparticipation.

### Measures

#### Ability self-concept

SC of one’s own abilities was assessed for each of the three applied psychological domains and for psychology courses in general. Three items from the Self-Description Questionnaire III (SDQ III; [68]), which is frequently used in SC research [69], were adapted to the context of psychology courses. Note that short forms represent valid alternatives for assessing SC in educational settings [70]. Participants rated their SC for each of the three psychological domains (e.g., “I’m good at *psychological domain*”) on a 6-point Likert scale, ranging from 1 (*not at all true*) to 6 (*completely true*). Additionally, three items assessed general SC (e.g., “I’m good at psychology”). The wording of items was parallel for each SC measurement. Items assessing SC are shown in Table 1.

**Table 1:** Items used for assessing ability self-concept and self-efficacy in psychology students.

Code	Self-concept
I/C/E 1	“I get good marks in *psychological domain*”
I/C/E 2	“I learn things quickly in *psychological domain*”
I/C/E 3	“I am good at *psychological domain*”
G 1	“I get good marks in psychology”
G 2	“I learn things quickly in psychology”
G 3	“I am good at psychology”
Code	Self-efficacy
I/C/E/G 4	When I am confronted with a problem in performing the given tasks, I can usually find several solutions.
I/C/E/G 5	I feel prepared for most of the demands.
I/C/E/G 6	I can remain calm when facing difficulties in performing the given tasks because I can rely on my abilities.
I/C/E/G 7	My past experiences in my studies have prepared me well for the given tasks.

I = Industrial and Organizational psychology; C = Clinical psychology; E = Educational psychology; G = General

#### Self-efficacy

SE is typically assessed in the context of performing specific tasks within a particular domain [41, 65]. Since it is not possible to assess every single task that a psychology student must master within the context of his or her university studies, we used carefully constructed and realistic scenarios (i.e., vignettes) of psychological tasks. Separate vignettes were presented for each of the three psychological domains (i.e., industrial and organizational psychology, clinical psychology, educational psychology). The vignettes referred to psychological tasks students have to cope with in their university studies and post-graduate jobs. To construct vignettes, we conducted interviews with experts and analyzed the study curricula (e.g., handbooks of study modules). Afterwards, experts reviewed the adequacy of each vignette. For an example, see Tables A1-A3 in S1 Table. Participants read the vignette and then responded to four SE items taken from an established scale by Rigotti et al. [71] (e.g., “I feel prepared for most of the demands”) on a 6-point Likert scale ranging from 1 (*not at all true*) to 6 (*completely true*). Students rated their SE separately for the described tasks in each psychological domain. Additionally, after having presented the vignettes for psychological tasks in the three domains, participants rated their SE to successfully master psychological tasks in general. Items assessing SE are shown in Table 1.

### Statistical analysis

To pool data, we first tested for measurement invariance of scales across undergraduate and graduate psychology students as well as across the paper-and-pencil and web measurements. Only if it can be shown that the scales operate comparably across assessment conditions (i.e., paper-pencil and web) and level of studies (i.e., undergraduate and graduate) is it legitimate to pool the data. All confirmatory factor analysis (CFA) models were conducted with Mplus Version 7 [72] using the maximum likelihood estimator (MLR), which is robust against mild violations of non-normal data distributions. For the test of measurement invariance, we started with the least constrained solution and successively imposed restrictions for equality of specific parameters (e.g., factor loadings) across groups to produce nested models, which were tested against each other using the chi-square test [73]. We further assessed model fit by evaluating other fit indices that are less sensitive to sample size than chi-square (e.g., ΔCFI; [74]). A change of ≥ -.010 in the comparative fit index (CFI) supplemented by a change of ≥ .015 in the root mean square error of approximation (RMSEA) indicates noninvariance [75]). To handle missing data, full information maximum likelihood (FIML) was used. The rate of missing data for the items measuring SC in industrial and organizational psychology, clinical psychology, educational psychology, and in psychology in general ranged from 7.4% to 12.4%. The rate of missing data for the items measuring SE in these three domains and in psychology in general ranged from 9.4% to 10.7%.

**Fig 1:**
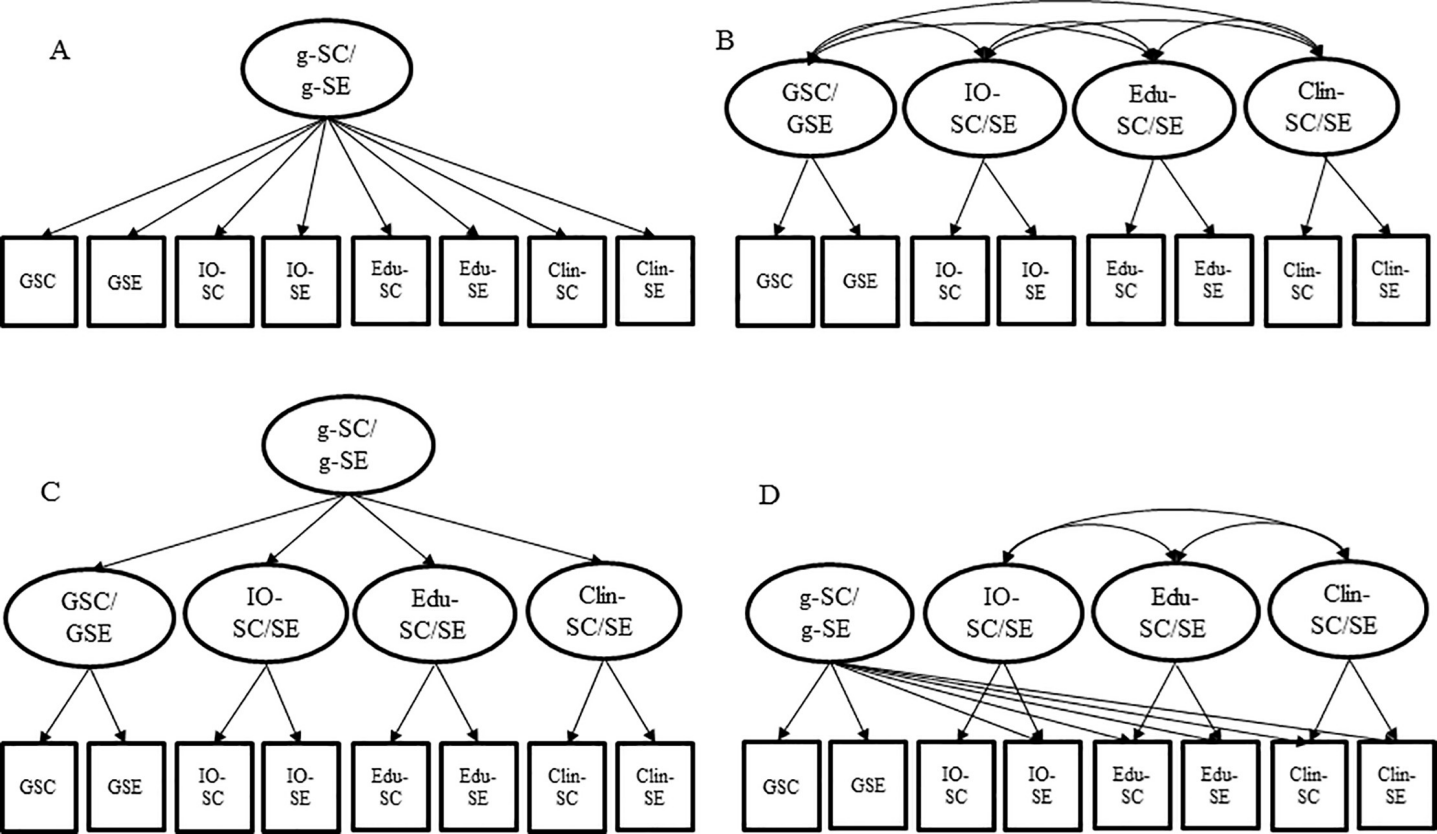
A-D. Four separate structural structural models of ability self-concept and self-efficacy in higher education. SC = Ability self-concept; SE = Self-efficacy; IO = Industrial and Organizational psychology; Edu = Educational psychology; Clin = Clinical psychology; GSC = General self-concept; GSE = General self-efficacy; g = General factor.

First, to provide the basis for an empirical comparison of SC and SE in higher education, we tested four different CFA models in order to examine the internal structure of SC and SE separately (see Fig 1). Within the different structural models, we focused on three structural aspects: the multidimensionality, the hierarchy, and the existence of a general (*g*) factor (see Table 2).

**Table 2:** Characteristics of the four different structural models.

Model	Multidimensionality	Hierarchy	*g* factor
A: *g* factor			X
B: first-order correlated factor	X		
C: second-order factor	X	X	X
D: incomplete bifactor	X		X

Model A represents a *g* factor model with one general factor at the apex as has been proposed for SE by some researchers for SE (e.g., [67, 76]). Model B contains four mutually correlated first-order factors that correspond to SC or SE in the three psychological domains and in psychology in general. Thus, Model B incorporates the idea of multidimensionality that has been widely shown for students’ SC [3] and in a few cases for SE [e.g., 4]. However, the model does not explicitly incorporate the idea of a general SC or SE factor. Model C represents a second-order model with a general factor that accounts for the shared variance of all domain-specific first-order factors [77]. Thus, Model C incorporates the idea of multidimensionality (first-order factors) and the idea of a hierarchically superordinate *g*-factor (second-order factor). Lastly, in Model D, in accordance with the nested Marsh/Shavelson model of students’ SC [3], we tested an incomplete bifactor model [78]. In contrast to the second-order model (Model C), in Model D the general SC/SE factor is not added as a hierarchically superordinate second-order factor, but directly accounts for the common variance in all manifest measures while being defined by the general items. Hence, the incomplete bifactor model separated from the item variance attributable to a *g*-factor from the variances attributable to domain-specific factors.

In all models (one for SC and one for SE), general and domain-specific SC and SE factors were specified as latent variables. The unstandardized loading of the first item of each SC/SE factor was fixed to 1 (Model A through Model D). Additionally, correlated uniqueness was allowed in all CFAs to account for the shared variance due to the common measurement method (i.e., common item wording). To evaluate model fit, we used the chi-square (χ2) goodness-of-fit statistic. Because this statistic is sensitive to sample size, we also used the following recommended descriptive measures of model fit [79]: (1) RMSEA, which should be below .06, (2) the comparative fit index (CFI), which should exceed .95, and (3) the standardized root mean square residual (SRMR), which should be below .08. Additionally, we evaluated relative model fit indices of the Akaike information criterion (AIC) and the Bayesian information criterion (BIC), for which lower values denote better model fit [79].

**Fig 2:**
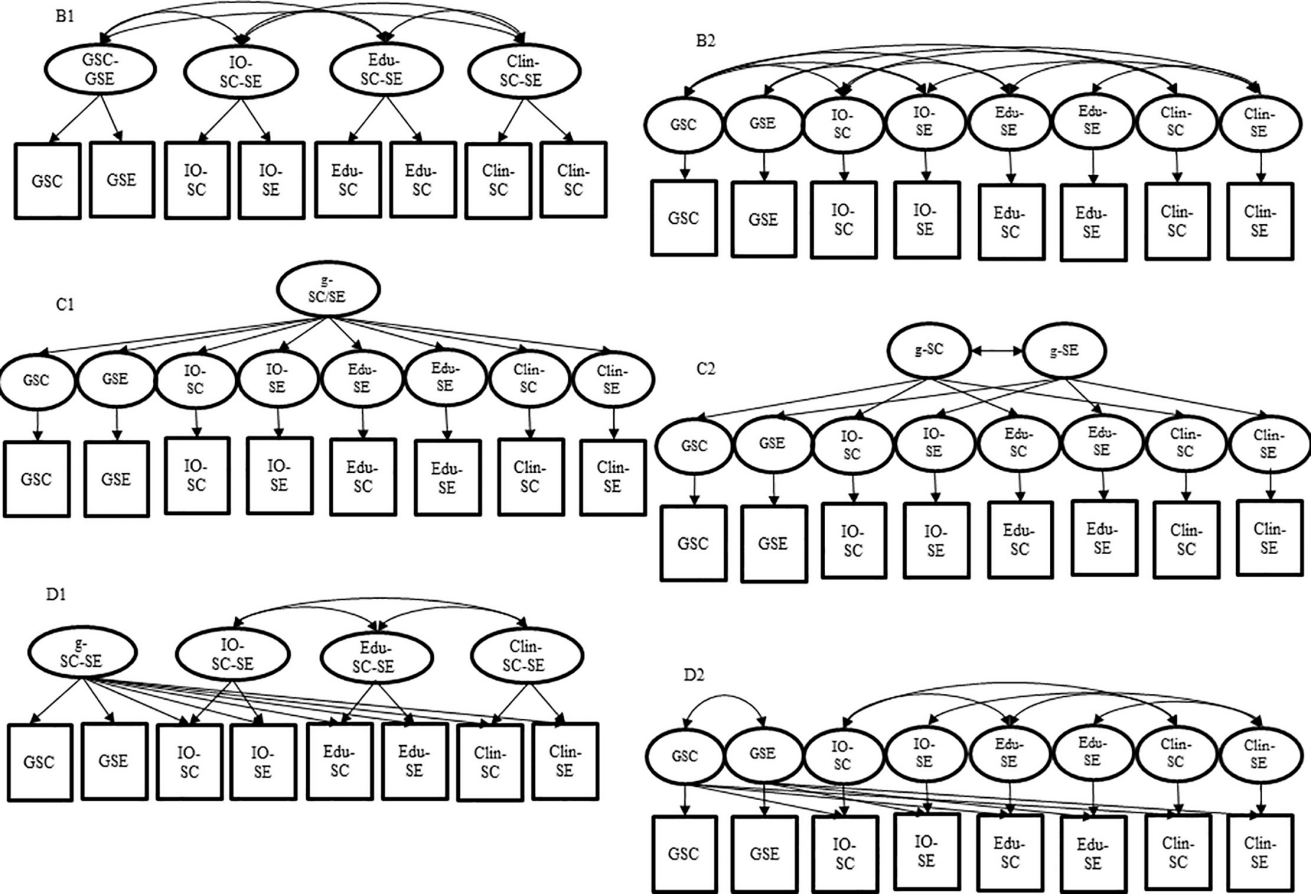
B1-D2. Six different combined structural models of ability self-concept and self-efficacy in higher education. SC = Ability self-concept; SE = Self-efficacy; IO = Industrial and Organizational psychology; Edu = Educational psychology; Clin = Clinical psychology; GSC = General self-concept; GSE = General self-efficacy; g = General factor.

Second, based on the findings of the first step, we investigated the empirical differentiation of SC and SE. We combined the models for both constructs (combined Models B-D; Model A was excluded due to poor model fit; see below) and either modeled SC and SE as common factors (B1-D1) or as separate SC and SE factors (B2-D2). The fit of the respective Models B to D with separate SC/SE factors was compared to the fit of the same model with combined SC/SE factors. For an overview of the different models, see Fig 2. Evaluation of model fit was conducted using the same absolute and incremental fit indices as for evaluating the fit of each separate structural model in the first step.

## Results

All descriptive statistics of the scales are shown in Table A4 in S1 Table. The scales used to assess SC and SE in the three different psychological domains and in general showed good to excellent reliabilities (αs = .856 to .951).

Results for the measurement invariance tests of the SC and SE scales across undergraduate/graduate students and paper-pencil/web measurement are reported in Tables A5-A6 in S1 Table. The evaluation of model fit indices and difference-testing statistics between more and less restricted models suggested that, in both cases, a model with partial scalar measurement invariance provided a good approximation to the data.

Table 3 gives an overview of the four structural models for SC and SE and their corresponding goodness-of-fit indices.

**Table 3:** Goodness-of-fit indices of alternative CFA models for ability self-concept and self-efficacy.

Model	*χ*^*2*^	*df*	CFI	TLI	RMSEA	SRMR	AIC	BIC
Self-concept								
A: *g* factor	3114.937	36	.673	.400	.267	.089	37446	37721
B: first-order correlated factor	177.975	30	.984	.965	.064	.030	33108	33413
C: second-order factor	189.181	32	.983	.966	.064	.033	33116	33411
D: incomplete bifactor	128.583	24	.989	.969	.060	.022	33057	33393
Self-efficacy								
A: *g* factor	2988.225	80	.742	.613	.179	.067	49039	49402
B: first-order correlated factor	268.953	74	.983	.972	.048	.035	45790	46183
C: second-order factor	277.481	76	.982	.972	.048	.035	45797	46180
D: incomplete bifactor	261.248	65	.983	.968	.052	.034	45797	46235

CFA = confirmatory factor analysis; χ^2^ = chi-square for all models is *p* < .001; df = degrees of freedom; CFI = comparative fit index; TLI = Tucker–Lewis index; RMSEA = root mean square error of approximation; SRMR = standardized root mean square residual; AIC = Akaike information criterion; BIC = Bayesian information criterion.

Evaluating the separate structural models of SC in regard to alternative fit indices, the *g* factor model (Model A) showed the lowest incremental and absolute fit (χ2 = 3114.937, *p* < .001, CFI = .673, TLI = .400, RMSEA = .267, SRMR = .089). The first-order correlated factor model (Model B) revealed good incremental and absolute model fit indices (χ2 = 177.975, CFI = .984, TLI = .965, RMSEA = .064, SRMR = .030) and lower and therefore better relative fit compared to the *g* factor model (Model A). The second-order model (Model C; χ2 = 189.181, *p* < .001, CFI = .983, TLI = .966, RMSEA = .064, SRMR = .033) showed a better model fit than the *g* factor model (Model A), but not compared to the first-order correlated factor model (Model B). In particular, the first-order-correlated factor model (Model B) represented the structure of SC slightly better than the second-order model (Model C). Lastly, Model D (incomplete bifactor model) with correlated domain-specific SC factors nested under a general SC factor resulted in the best fitting model (χ2 = 128.583, *p* < .001, CFI = .989, TLI = .969, RMSEA = .060; SRMR = .022). For Model B through Model D, all incremental and absolute fit indices fulfilled the conventional benchmarks [74], while AIC (33057) and BIC (33393) of the incomplete bifactor model had the lowest (i.e., best) values of all four models.

For SE, the *g* factor model (Model A) showed the lowest incremental and absolute fit (χ2 = 2988.225, *p* < .001, CFI = .742, TLI = .613, RMSEA = .179, SRMR = .067). The first-order correlated factor model (Model B) revealed good incremental and absolute model fit indices (χ2 = 268.953, *p* < .001, CFI = .983, TLI = .972, RMSEA = .048, SRMR = .035) and lower relative fit compared to the *g* factor model (Model A). As for SC, the second-order model of SE (Model C; χ2 = 277.481, *p* < .001, CFI = .982, TLI = .972, RMSEA = .048, SRMR = .035) showed a better model fit than the *g* factor model (Model A), but not compared to the first-order correlated factor model (Model B). In particular, the first-order-correlated factor model (Model B) represented the structure of SE equally well as the second-order model (Model C). Lastly, Model D (incomplete bifactor model), with correlated domain-specific SE factors nested under a general SE factor, resulted in an equally good fitting model (χ2 = 261.248, *p* < .001, CFI = .983, TLI = .968, RMSEA = .052; SRMR = .034) as Model B and C. As for SC, for Models B through D, all incremental and absolute fit indices fulfilled the conventional benchmarks [74], and AIC (52065) and BIC (52679) had comparably high values for all three models. Factor loadings of all eight models for SC and SE are reported in Tables A7-A9 in the S1 Table.

Based on the fit indices of the separate models, we chose the first-order correlated factor model (Model B), the second-order factor model (Model C), and the incomplete bifactor model (Model D) since all three have a comparably good fit for SC and SE. We synthesized them into combined models of SC and SE (Models B1 to D2, for results see Table 4). Note that we decided not to investigate the *g* factor model in a second step due to poor model fit indices in the first step (see Table 3).

**Table 4:** Goodness-of-fit indices for the different combined models.

Model	*χ*^*2*^	*p*	*df*	CFI	TLI	RMSEA	SRMR	AIC	BIC
B1: Combined first-order factor model with 4 factors	4718.768	< .001	302	.886	.857	.087	.106	80159	80832
B2: Combined first-order factor model with 8 factors	700.731	< .001	280	.983	.976	.035	.033	77291	78077
C1: Combined second-order factor model with one second-order *g* factor	1610.344	< .001	300	.946	.932	.060	.062	78372	79056
C2: Combined second-order factor model with two correlated second-order *g* factors	1413.095	< .001	299	.954	.942	.055	.052	78125	78814
D1: Combined incomplete bifactor model with one *g* factor	3142.238	< .001	285	.882	.843	.091	.108	80257	81017
D2: Combined incomplete bifactor model with two correlated *g* factors	638.959	< .001	265	.985	.978	.034	.031	77244	78106

*χ*^*2*^ = chi-Square; *df* = degrees of freedom; CFI = comparative fit index; TLI = Tucker–Lewis index; RMSEA = root mean square error of approximation; SRMR = standardized root mean square residual; AIC = Akaike information criterion; BIC = Bayesian information criterion.

Model B1 (first-order correlated factor model) had four different first-order factors, that is, one common SC and SE first-order factor for each psychological domain and for psychology in general. Model B2, in contrast, had eight factors: one first-order factor for each psychological domain and each construct of SC and SE (e.g., one factor for SC in clinical psychology and one factor for SE in clinical psychology). In both Models B1 und B2, the domain-specific factors were allowed to correlate within and across constructs. Results revealed, that the combined first-order correlated factor model with four factors (Model B1) had a worse fit than the model with eight correlated factors (Model B2; *χ*^*2*^ = 700.31, *p* < .001, CFI = .983, TLI = .976, RMSEA = .035, SRMR = .033).

Because Model B1 had a worse model fit than Model B2, we used Model B2 as base for Models C1 and C2. That is, Model C1 assumed one second-order factor which accounts for the common variance of the eight first-order factors, whereas in Model C2, there were two different but correlated second-order factors (one for the four first-order factors of SC and one for the four first-order factors of SE). The combined second-order factor model with two correlated factors (Model C2; *χ*^*2*^ = 1413.095, *p* < .001, CFI = .954, TLI = .942, RMSEA = .055, SRMR = .052) had a slightly better fit than the combined second-order factor model with only one general factor (Model C1).

Finally, Model D1 included four first-order factors representing common SC and SE factors in the three psychological domains and in psychology in general. Further, the model included only one *g* factor that accounts for the common variance of all measures of SC and SE while being defined by the general items. Model D2 had eight separate first-order factors for SC and SE, and two correlated *g* factors representing either general SC or general SE. In both models, D1 und D2, the domain-specific factors were allowed to correlate within and across constructs. Further, the general SC factor could correlate with the general and domain-specific SE factors and vice versa. The combined incomplete bifactor model, with eight first-order factors and two correlated *g* factors (Model D2), showed a better model fit (*χ*^*2*^ = 638.959, *p* < .001, CFI = .985, TLI = .978, RMSEA = .034; SRMR = .031) than Model D1, with only four first-order factors and only one single *g* factor.

Overall, next to Model B2, Model D2 comparatively showed the best fit, even if only marginal. Table 5 shows the factor correlations for this model (for the correlations within Model B1, B2 and D1, see Tables A10-A12 in the S1 Table). Within Model D2, the latent correlation between the *g* factors of SC and SE was *r* = .604, *p* < .001. The correlations between the SC factors of the three psychological domains ranged between *r* = .535, *p* < .001 and *r* = .710, *p* < .001; for SE factors, the range was considerably lower (*r* = .367, *p* < .001 to *r* = .499, *p* < .001). Correlations between constructs within a domain (e.g., between SC and SE in educational psychology) ranged between *r* = .565, *p* < .001 and *r* = .680, *p* < .001. Factor loadings of the six combined models are reported in Tables A13-A17 in the S1 Table.

**Table 5:** Correlations between ability self-concept and self-efficacy factors within the combined incomplete bifactor model with eight first-order factors and two *g* factors (Model D2).

	SC IO	SC Clin	SC Edu	SC Gen	SE IO	SE Clin	SE Edu
SC IO	-						
SC Clin	.535***	-					
SC Edu	.560***	.710***	-				
SC Gen	-	-	-	-			
SE IO	.680***	.276***	.355***	.027	-		
SE Clin	.305***	.624***	.408***	.003	.367***	-	
SE Edu	.286***	.367***	.565***	.049	.446***	.499***	
SE Gen	.230***	.254***	.213***	.604***	-	-	-

SC = Ability self-concept; SE = Self-efficacy; IO = Industrial and organizational psychology; Clin = Clinical psychology; Edu = Educational psychology; Gen = General.

*p < .05. **p < .01.

***p < .001.

## General discussion

In the present study, we tested the empirical differentiation of SC and SE in a large sample of psychology students. By doing so, we tested the generalizability of previous findings regarding the empirical differentiation of SC and SE from K-12 educational settings to higher education in the specific discipline of psychology. In order to provide the base for such an investigation, we analyzed the factorial structure of psychology students’ SC and SE using only cognitive SC items and measuring both constructs at the same level of specificity in different domains and at different levels of hierarchy. Findings revealed that psychology students’ SC and SE are both multidimensional, including domain-specific SC or SE factors as well as a general SC or SE factor. We found no clear results with regard to the hierarchy of both constructs because, for both SC and SE, the first-order correlated factor model and the incomplete bifactor model showed a comparable fit as did the second-order model. Based on these three well-fitting factorial models, we integrated SC and SE into different combined structural models that accounted for the factorial structure of both constructs simultaneously. Within these combined models, we investigated the empirical differentiation between SC and SE by analyzing model fit indices and correlations between SC and SE factors at a domain-specific level and at a general level. Results revealed that a combined first-order factor model with eight first-order factors (Model B2) and a combined incomplete bifactor model with eight first-order factors and two *g* factors (Model D2) best explained the data. Within these models, domain-specific or general SC and SE were significantly and positively correlated within each domain and at each level of specificity. Both combined models supported the empirical differentiation of SC and SE as two positively correlated but empirically separable constructs.

Our findings add empirical evidence that SC and SE are positively correlated but distinct constructs in higher education, and they support the generalizability of findings from the K-12 school system [12, 13] to the specific discipline of psychology in higher education. Note that both educational settings have much in common, for example, allowing students to integrate achievement-related information from different sources to form their SC and SE or to experience academic successes and disappointments that alter SC and SE [19]. Thus, our research findings can be used to learn about generalizability of findings to further academic domains or disciplines in higher education.

With regard to the structure of psychology students’ SC, the finding of a multidimensional structure is well aligned with findings from K-12 educational settings [e.g., 54, 55]. All models in our study, except for the *g* factor model, assume a multidimensional structure of SC in line with the assumption of the SC structure proposed by Shavelson et al. [53]. Besides multidimensionality, the results also supported the notion of a general SC for the sample of psychology students. This finding is again in line with findings from the K-12 educational context, where there is ample evidence for the multidimensionality of SC with a general SC factor at the apex [58, 59].

Our results further revealed that assumptions about multidimensionality also apply to psychology students’ SE. This finding contributes essentially to the debate on the structure of SE. Despite different views on the structure of SE [64–66, 80], researchers have not developed and investigated different structural models of SE [6]. In the present study, the first-order factor model, the second-order model, and the incomplete bifactor model provided a good fit, representing the different perspectives on the structure of SE. In previous research, some authors found evidence for a general SE construct [5], whereas others found evidence only for multidimensionality but not for hierarchy of SE [4, 77]. Our analytical procedure of beginning with structural analyses of SE might, therefore, be a good starting point for further investigations of the question of the factorial structure of SE in diverse educational settings with additional samples, such as school students or university students enrolled in different study programs. To conclude, we found that all three models (i.e., Models B-D) fit well for SC and SE. The choice of a specific model, however, does not only depend on model fit indices but also on theoretical considerations and the respective research question. If, for example, one would like to make a statement about the SC or SE profile of a student, the first-order correlated factor model seems to be the most appropriate model, since it represents domain-specific characteristics, and thus enables statements to be made about domain-specific strengths and weaknesses. The incomplete bifactor model, in contrast, is useful when answering questions of conceptual closeness of domains by separating the variance of domain-specific factors from that of the general factor.

With regard to the main question of empirical differentiation of SC and SE in our higher education sample, there has been a recent trend toward measuring SE at a more general level than the task level (e.g., at a domain-specific level; [64]). Note that SC items usually refer to specific domains, whereas SE items typically refer to specific tasks [3]. Similar to a study by Pietsch et al. [23], we also measured SC and SE at comparable levels of specificity (domain-specific and general), using only cognitive items. With regard to the aspect of specificity, first note that there are two types of specificity of self-perceptions that should be taken into account when comparing SC and SE empirically, namely domain specificity and measurement specificity [20]. The first one is related to the distinction of self-perceptions across different domains (e.g., clinical psychology and educational psychology); the second one emphasizes the level at which these self-perceptions are measured within domains (e.g., task-specific or domain-specific). Previous research has not controlled for domain and measurement specificity simultaneously when comparing SC and SE [12, 13, 20, 23]. Moreover, the domain-specific and general conceptualizations of SC and SE have not been explored simultaneously in previous research. Hence, the reported differences between SC and SE of previous studies should be interpreted with caution. We, however, in contrast to, for example, Pietsch et al. [23], integrated both constructs in different combined models in consideration of their internal structure before we compared SC and SE empirically. We found evidence for empirically distinct domain-specific and general factors of SC and SE as indicated by good model fit of the combined first-order correlated factor model with eight correlated first-order factors and the combined incomplete bifactor model with two correlated *g* factors.

However, in line with previous studies [12–14, 16, 51], we found large positive correlations between SC and SE at general and domain-specific levels, thus, both constructs have much in common, for example, their emphasis on competences [8]. Hence, although students’ SC is mostly formed through comparison processes and SE through mastery experiences [6], the same previous experience in a specific domain or in general can provide valid information for both competence-related self-perceptions [2]. The stronger overlap of SC and SE within domains compared to the correlations across domains is a further indication of the comparable multidimensional structure of both constructs. Building on this, the question arises whether both constructs are not only correlated, but also related reciprocally within domains. In this regard, Ferla et al. [11] assumed that students who believe to be able in mathematics (SC) should subsequently feel more SE to master specific mathematic tasks that in turn again should foster their mathematic SC. If the consideration of a reciprocal relation between SC and SE might be true, SC in clinical psychology, for example, influences the development of one’s expectancy beliefs for success on a specific future task in clinical psychology (SE) [17] and vice versa. Concluding, both constructs are mutually dependent, but distinguishable. In the K-12 educational setting, when discussing the factorial structure of SC, researchers have investigated how subjects can be arranged on a continuum (e.g., [59, 81]). When looking at the cross-domain correlations in the present study (see Table 5), we found higher correlations between SC in clinical psychology and SC in educational psychology than between SC in clinical psychology/SC in educational psychology and SC in industrial and organizational psychology. On a domain-based continuum, psychology students might perceive the domain of industrial and organizational psychology at greater distance from the two other psychological domains. However, further research on the arrangement of domains is needed.

## Limitations

Despite its strengths, it should be kept in mind that the present study has some limitations. First, even though we believe that our results on the factorial structure of psychology students’ SC and SE can be generalized to other German psychology students, the sample was not representative, especially regarding student populations outside of Germany. Besides, we do not know whether SC and SE of university students in different study programs (e.g., business) also form a multidimensional and to some extent hierarchical structure as found for students of psychology. Moreover, student populations from majors other than psychology might have might have less professional knowledge or professional training concerning the constructs (SC and SE) and may be less capable of responding to these items than students of psychology. This also restricts the generalizability of the results to further student populations. However, our results contribute to the analysis of SC and SE in higher education and form a basis for further structural examinations of both constructs with diverse student samples in different countries.

Second, we did not compare SC and SE empirically by investigating their predictive power for external criteria, such as achievement indicators (grades in undergraduate/graduate psychology courses) as several researchers have done (e.g., [12, 20]). The reason for this is that we only had self-reported grades in our data set. Even if grades are useful achievement indicators, we must question their reliability and validity, in particular if they serve as the only achievement indicator [82]. Moreover, grades are difficult to compare in the higher education context, for example, with regard to the large number of different courses for which we cannot control the influence of social comparison processes on the SC developmental process.

Third, we only investigated the differentiation of SC and SE in three applied subjects. Note that within the German university psychology curriculum, next to the applied psychological domains, statistics, multivariate methods, applied assessment, and evaluation are treated as separate subjects, which are taught in separate study modules. However, statistical research methods are also included in the applied psychological subjects with regard to specific tasks (e.g., constructing a measurement instrument in educational psychology). Thus, German psychology students must master specific statistical tasks in different psychological domains, so that the individual confidence to successfully deal with those tasks might generalize over the different domains. This task-related generalization across domains could be represented in a *g* factor. Future studies should therefore add the domain of statistics when investigating the structure of SC and SE of psychology students to investigate whether this domain can explain a certain proportion of variance in a *g* factor.

Fourth, measures of SC and SE were obtained from a single source (self-report). This measurement strategy potentially makes the results susceptible to method bias [83], although it should be noted that meta-analytic results support the validity of self-report measures [84] and that SC and SE are usually assessed by self-report.

Fifth, scalar measurement invariance of the scales (which is a precondition to compare factor means across undergraduate and graduate psychology students) was only partially established in the three psychological domains. One possible explanation is that students can already assess their abilities in a more differentiated way in the graduate program compared to students in the undergraduate program. Multidimensionality is thus even more clearly developed. The aspect of required measurement specificity brings up another limitation. We investigated the structure of SC and SE at domain-specific and general levels, but we did not investigate the task-specific structure of SC and SE. Consequently, we were not able to compare both constructs at that level of hierarchy. Moreover, we do not know whether it is possible to measure SC at a task-specific level [8, 20, 50], thus, at which levels SC and SE can be operationalized and assessed remains an unresolved question (e.g., [7]).

## Implications and future research

In the present study, only the cognitive component of SC was considered and compared with SE to prevent confounding by the inclusion of affective components in the SC measure [18]. In the future, the affective component should also be recorded (e.g., “I like educational psychology”) to replicate the two-component structure of SC for this higher education context. Based on their internal structure, future research should further compare the predictive power of SC (affective and cognitive) and SE measures with, for example, academic achievement, effort, and coursework selections matched in specificity [14, 22, 85] within domains to investigate the incremental validity of SC over SE and vice versa. Studies with students from Western countries found evidence for a positive relationship between the cognitive component of SC and SE with academic achievement within domains [e.g., 13, 35], whereas the affective component of SC is related to domain-specific affective outcomes such as academic interest ([86]). Surprisingly, for students from Eastern cultures, researchers found evidence for opposite correlations. For example, Yang [87] reported evidence for a stronger relationship between the affective (instead of the cognitive) component of SC with matching domains of academic achievement for Chinese students [see also [88, 89]. Concluding, future research should integrate samples from different educational contexts and diverse cultures to investigate the predictive power of SC, its components, and SE.

If studies will find that SC or SE predicts academic outcomes better than the other construct, this provides further evidence that SC and SE represent different constructs [9]. Empirical comparisons of the predictive power of SC and SE should also include further related constructs such as self-esteem, outcome expectations, or self-confidence. Note that many items begin with the stem “I’m confident” [65]. However, according to Stankov and Lee [90], SE should better be measured by questions like “I can do this” in relation to a future task, instead of asking people for their subjective confidence to correctly master a specific task [6]. Such multivariate investigations help to minimize the risk of *jingle-jangle* fallacies [6, 91]. Thus, significant contributions can be made to science by simultaneously analyzing psychological constructs that are considered to be different (e.g., concerning their relative predictive power). We suggest to start such a comparison with a structural analysis of the constructs of interest.

Additionally, longitudinal studies would be useful to compare the within-domain and cross-domain relations between SC, SE, and achievement in different domains and in general. In the K-12 educational setting, there is ample evidence for reciprocal relations between SC and achievement [27, 32], but less evidence concerning the longitudinal relation between SE and achievement [92, 93]. Longitudinal studies also provide the basis for investigating the development of SC and SE. In the K-12 educational context, it is known that SC achieves multidimensionality at an early stage and then remains stable [18]. To date, there are hardly any findings on the development of domain-specific SE factors. Additionally, the question of how the *g* factor of both constructs emerges and how it develops over time should be part of future studies.

In addition to theoretical implications, our findings also have practical relevance. The knowledge about the internal structure of psychology students’ SC and SE can help us to develop domain-specific intervention strategies [94, 95]. However, as SC and SE are highly and positively correlated, and in our study we have no criterion variable to support their empirical difference, the practical relevance of the differentiation of both constructs within psychology studies is only tentative.

The most important source of SC are processes of comparison as described in the internal/external frame of reference model [30]. Instructors should guide students to reflect and relativize a possible worse evaluation of their own performance in one domain to minimize negative cross-domain effects caused by dimensional comparison processes and to develop a realistic self-view. Since the self-assessment of one's own abilities is closely related to academic and occupational choice [83, 96], a realistic self-perception supports the career development of students [97], guaranteeing students’ employability [98]. It also makes sense for instructors to use not only grades as performance feedback, but also open performance feedback (e.g., of a student's work behavior over the semester to relativize the informative power of a single grade). Additionally, students who reflect on their abilities and on their individual learning/development process (source of mastery experiences) should perform better than those who do not reflect on their abilities [99]. Instructors could therefore induce a steady reflection and judgment of one’s own acquired abilities in several academic domains (e.g., keeping a learning diary). Besides, interventions could also make use of attributions that accompany a positive SC (e.g., attributions to abilities in success situations; [100]). Finally, one could also make use of subjective values in enhancing university students’ SCs by implementing *Utility Value Interventions* [101]. According to these interventions, instructors asks students to explain how the learning material is relevant to their lives. The individual importance and value that students attribute to a specific domain (e.g., educational psychology) subsequently leads to more interest, better performance, and positive SC in this domain [101].

As for SC, university students’ SE can also be enhanced by supporting the different major sources of SE (e.g., Phan [102]; for an overview, see [94]). With the focus on the source of vicarious experiences, Adams [103] used a small case study with counseling students to compare the influence of observing a seminar presentation of a peer versus of a senior academic on SE of participants. Results indicated that observing a peer model had more potential to strengthen the SE of the participants, due to a greater similarity between them and the role models. In the university setting, especially fellow students could act as role models, whereby instructors should emphasize similarities between themselves and the students. The source of verbal persuasion (e.g., “*You can do this”*, [5], p. 160) can also be useful to strengthen the SE of university students. For example, in higher education, this could be accomplished by performance feedback from fellow students who are viewed as knowledgeable and credible [2]. Usually, the most powerful basis upon which students can judge their own abilities are past performances (mastery experiences). The method of reflection not only fosters individuals’ SC, but also makes one aware of his or her abilities and positive mastery experiences so that one feels more self-efficacious when faced with the same challenging tasks in the future. Analog to an experiment by Dempsey and colleagues [104], psychology students should receive performance feedback based not only on grades, but also on their performances in different tasks during the semester in, for example, seminars and tutorials [105].

Overall, there is a vast body of intervention methods that positively affect sources of SC or SE [92, 106–110]. Based on the knowledge of their factorial structure, future research can investigate diverse intervention methods to support students’ SC and SE.

## Conclusion

The analysis of the factorial structure of SC and SE is a central presupposition to investigate their empirical differentiation with each other and with further constructs and criteria in educational settings. Our results show that when investigating the factorial structure of SC and SE considering a broader range of domains, both constructs can be measured at comparable levels of specificity. Additionally, using only cognitive SC items, SC and SE in higher education (e.g., of psychology students) form positively related but empirically separable constructs at domain-specific and general levels. However, more research is needed to extend the understanding of the empirical separation of SC and SE to further populations of university students in different countries, including tests of their predictive validity for various academic outcomes such as academic achievement and development.

## Supporting information

S1 Table Additional tables. (DOCX)

S1 Data SPSS dataset. (SAV)
