# Supplementary material for: Ability self-concept and self-efficacy in higher education: An empirical differentiation based on their factorial structure
Source: PLoS One. 2020 Jul 21;15(7):e0234604. doi: 10.1371/journal.pone.0234604 (PMC7373275; doi:10.1371/journal.pone.0234604)
Supplement: S1 Table — (DOCX) [file pone.0234604.s001.docx]

**Table A1.Vignette describing the factors of diagnostic, intervention, and research/evaluation in the domain of industrial and organizational psychology.**

| Imagine that you are working in the field of industrial and organizational psychology. Work demands comprise tasks in diagnostics, interventions and research/ evaluation. In diagnostics, you are working for an employer who specializes in personnel and organizational diagnostics. In your work, you are involved in the assessment and analysis of personnel selection, personnel development, and organizational development. For example, you will assess variables such as leadership skills, teamwork ability, job satisfaction, job motivation, or workload and stress factors. As methods you use questionnaires, standardized interviews, systematic behavioral observation, and psychological tests.  In interventions, you are working for an employer who specializes in consulting organizations and implementing interventions in the field of human resources and organizational development. This includes, for example, advanced training courses for both employees and managerial staff as well as seminars on the subject of time management and the corresponding training courses. Further examples of interventions are optimization measures for the design of workplaces and workflows as well as for team processes.  In research/evaluation, you are working for an employer who specializes in research and evaluation in the field of industrial and organizational psychology. You are involved in research and evaluation projects. In research projects, you gain new empirical insights based on previous research, for example, in the areas of teamwork, work motivation, and stress management. In evaluation projects, you examine the effectiveness of measures, for example, in the areas of communication, job safety, and teamwork. |
| --- |

**Table A2. Vignette describing the factors diagnostic, intervention, and research/evaluation in the domain of clinical psychology.**

| Imagine that you are working in the field of clinical psychology. Work demands comprise tasks in diagnostics, interventions and research/ evaluation. In diagnostics, you are working for an employer who specializes in the diagnosis of mental health problems. As part of your work you are involved in the classificatory, biographical, and functional assessment of indications as well as progression and process assessment. You are also responsible for evaluating the effectiveness of therapeutic measures. The mental problems range from anxiety disorders, eating disorders, and affective disorders to personality disorders. As methods you use standardized interviews and test procedures, systematic behavioral observation as well as guidance and counseling techniques.  In interventions, you are working for an employer who specializes in the preventative care of mental disorders, counseling those affected by mental health problems as well as recommending suitable therapies. In your work, you are involved in the counseling and adjustment processes of the affected persons, their family members, and other interested parties. This includes identifying and providing the appropriate therapy and promoting good health through preventative, supportive, and rehabilitative measures. For example, you are expected to counsel clients and their relatives in crisis situations or be able to intervene in case of relationship problems and social conflict situations.  In research/evaluation, you are working for an employer who specializes in research and evaluation in the field of clinical psychology. You are involved in research and evaluation projects. In the research projects, you gain new empirical knowledge based on the extant scientific research. This can include counseling and psychotherapy or specific disorders such as depression and schizophrenia. In evaluation projects, you examine the effectiveness of measures such as anti-smoking campaigns, new counseling techniques, or psychoeducational practices. |
| --- |

**Table A3. Vignette describing the factors assessment, intervention, and research/evaluation in the domain of educational psychology.**

| Imagine that you are working in the field of educational psychology. Work demands comprise tasks in diagnostics, interventions and research/ evaluation. In diagnostics, you are working for an employer who specializes in the prerequisites and outcomes of learning and achievement in the fields of education and training. In your work, you focus on the assessment of needs, constraints, and potentials of both students and teachers, on the identification of learning processes and outcomes, and on the evaluation of learning environment characteristics. As methods you use questionnaires, standardized interviews, systematic behavioral observations, and psychological test procedures.  In interventions, you are working for an employer who specializes in supporting students and teachers as well as assisting them in their educational activities. In your work, you are involved in counseling and training students, parents/guardians, families, teachers, and educational staff. This includes educational guidance, learning guidance, counseling of socioemotional problems in the educational context (e.g., bullying), as well as training of learning skills, parenting competence (e.g., parent training), or socioemotional skills (e.g., violence prevention).  In research and evaluation, you are involved in research and evaluation projects. In the research projects you gain new empirical knowledge on the basis of previous research, for example, in the areas of designing learning environments, assessing competences, or predicting performance. In evaluation projects, you examine the effectiveness of measures such as training programs to promote social skills or methods for optimizing learning environments |
| --- |

**Table A4. Descriptive statistics of the test scales (N = 1243).**

|  | *N* | *M* | *SD* | Min | Max | $\alpha$ |
| --- | --- | --- | --- | --- | --- | --- |
| Self-concept Industrial and Organizational  Self-concept Clinical psychology | 1101  1107 | 3.19  3.63 | 1.41  1.49 | 1.00  1.00 | 6.00  6.00 | .941  .951 |
| Self-concept Educational psychology | 1111 | 3.50 | 1.41 | 1.00 | 6.00 | .947 |
| Self-concept General | 1085 | 3.88 | 1.24 | 1.00 | 6.00 | .917 |
| Self-efficacy Industrial and Organizational  Self-efficacy Clinical psychology | 1113  1109 | 3.07  3.33 | 1.09  1.16 | 1.00  1.00 | 6.00  6.00 | .858  .868 |
| Self-efficacy Educational psychology | 1116 | 3.31 | 1.11 | 1.00 | 6.00 | .869 |
| Self-efficacy General | 1101 | 3.60 | 1.06 | 1.00 | 6.00 | .890 |

*Note*. *N* = sample size, *M* = means; *SD* = standard deviation; Min = Minimum; Max = Maximum; α = Cronbach's Alpha.

**Table A5. Measurement invariance of ability self-concept scales among undergraduate and graduate psychology students and across paper and pencil and web questionnaire.**

|  | *χ2* | *df* | Model comparison | ΔSB*χ2* | Δdf | CFI | ΔCFI | RMSEA | Δ RMSEA |
| --- | --- | --- | --- | --- | --- | --- | --- | --- | --- |
| undergraduate/ graduate | | | | | | | | | |
| SC Industrial, and Organizational psychology | | | | | | | | | |
| configural (c)  metric (m)  scalar (s)  partial^a^ scalar (ps) | 0.00  1.327  79.609***  3.114 | 0  2  4  3 | -  m-c  s-m  ps-m | 0.00  1.33  86.26***  1.84 | 0  2  2  1 | 1.00  1.00  .917  1.00 | -  .00  -.083  .000 | 0.00  .00  .182  .008 | -  .00  .182  .008 |
| SC Clinical psychology | | | | | | | | | |
| configural (c)  metric (m)  scalar (s)  partial^b^ scalar (ps) | 0.00  3.225  30.597***  11.252 | 0  2  4  3 | -  m-c  s-m  ps-m | .00  3.23  30.62***  9.53* | -  2  2  1 | 1.00  .999  .974  .992 | -  -.001  -.025  -.007 | 0.00  .033  .108  .069 | -  .033  .075  .036 |
| SC Educational psychology | | | | | | | | | |
| configural (c)  metric (m)  scalar (s)  partial^c^ scalar (ps) | 0.00  0.52  42.73***  0.67 | 0  2  4  3 | -  m-c  s-m  ps-m | 0.00  0.52  41.59***  0.14 | -  2  2  1 | 1.00  1.00  .130  1.00 | -  .00  .130  .00 | .00  .00  .964  .00 | -  .00  -.036  .00 |
| SC General | | | | | | | | | |
| configural (c)  metric (m)  scalar (s)  partial^d^ scalar (ps) | 0.00  1.80  19.40  1.95 | 0  2  4  3 | -  m-c  s-m  ps-m | 0.00  1.80  18.61***  0.11 | -  2  2  1 | 1.00  1.00  .987  1.00 | -  .00  -.013  .00 | .00  .00  .084  .00 | -  .00  .084  .00 |
| paper-pencil/ web | | | | | | | | | |
| SC Industrial, and Organizational psychology | | | | | | | | | |
| configural (c)  metric (m)  scalar (s)  partial^e^ scalar (ps) | 0.00  4.00  13.01  4.08 | 0  2  4  3 | -  m-c  s-m  ps-m | 0.00  4.00  8.56*  2.62 | -  2  2  1 | 1.00  .998  .990  .999 | -  -.002  -.008  .001 | 0.00  .042  .063  .025 | -  .042  .021  -.017 |
| SC Clinical psychology | | | | | | | | | |
| configural (c)  metric (m)  scalar (s) | 0.00  2.82  2.63 | 2  4  2 | -  m-c  s-m | -  2.82  0.07 | -  2  2 | 1.00  .999  1.00 | -  -.001  .00 | .00  .027  .00 | -  .027  .00 |
| SC Educational psychology | | | | | | | | | |
| configural (c)  metric (m)  scalar (s) | 0.00  4.12  7.19 | 2  4  2 | -  m-c  s-m | -  4.12  3.05 | -  2  2 | 1.00  .998  .997 | -  -.002  -.001 | .00  .043  .037 | -  .043  -.006 |
| SC General | | | | | | | | | |
| configural (c)  metric (m)  scalar (s) | 0.00  4.76  10.52 | 0  2  4 | -  m-c  s-m | -  4.58  5.75 | -  2  2 | 1.00  .098  .094 | -  -.002  -.004 | .00  .050  .055 | -  .050  .005 |

*Note.* SC = Ability self-conept; *χ2* = Chi-Square; *df* = degrees of freedom; CFI = comparative fit index; RMSEA = root mean square error of approximation; SB*χ2* = Satorra-Bentler scaled chi-square difference test; ΔCFI ≥ │.0.10│, ΔRMSEA ≥│.015│ signal lack of invariance between nested models; configural = factor loadings are invariant; metric = factor loadings and intercepts are invariant; scalar = factor loadings, intercepts, and residuals are invariant, partial scalar = factor loadings, and residuals are invariant, intercepts are partial invariant.

^a^Intercept I1 released.

^b^Intercept C1 released.

^c^Intercept E1 released.

^d^Intercept G1 released.

^e^Intercept I1 released.

**p* < .05. ***p* < .01. ****p < .*001.

**Table A6. Measurement invariance of self-efficacy scales among undergraduate and graduate psychology students and across paper and pencil and web questionnaire.**

|  | *χ2* | *df* | Model comparison | ΔSB*χ2* | Δdf | CFI | ΔCFI | RMSEA | Δ RMSEA |
| --- | --- | --- | --- | --- | --- | --- | --- | --- | --- |
| undergraduate/ graduate | | | | | | | | | |
| SE Industrial, and Organizational psychology | | | | | | | | | |
| configural (c)  metric (m)  scalar (s)  partial^a^ scalar (ps) | 44.72  57.63  76.26  65.10 | 4  7  10  9 | -  m-c  s-m  ps-m | 0.00  7.14  17.81***  5.45 | -  3  3  2 | .969  .962  .950  .958 | -  -.007  -.012  -.004 | .135  .114  .109  .105 | -  -.019  -.005  -.009 |
| SE Clinical psychology | | | | | | | | | |
| configural (c)  metric (m)  scalar (s)  partial^b^ scalar (ps) | 78.29  85.20  100.34  91.58 | 4  7  10  9 | -  m-c  s-m  ps-m | -  0.82  13.12**  4.98 | -  3  3  2 | .942  .939  .929  .935 | -  -.003  -.010  -.004 | .182  .141  .127  .128 | -  -.041  -.014  -.013 |
| SE Educational psychology | | | | | | | | | |
| configural (c)  metric (m)  scalar (s)  partial^c^ scalar (ps) | 61.55  74.34  88.75  76.59 | 4  7  10  8 | -  m-c  s-m  ps-m | -  2.68  12.61**  0.23 | -  3  3  1 | .959  .952  .944  .951 | -  -.007  -.008  -.001 | .160  .141  .118  .123 | -  -.019  -.023  -.018 |
| SE General | | | | | | | | | |
| configural (c)  metric (m)  scalar (s)  partial^d^ scalar (ps) | 75.40  88.65  112.79  92.126 | 4  7  10  8 | -  m-c  s-m  ps-m | -  6.77  22.05***  0.37 | -  3  3  1 | .942  .934  .917  .932 | -  -.008  -.017  -.002 | .179  .145  .136  .137 | -  -.034  -.009  -.008 |
| paper-pencil/ web | | | | | | | | | |
| SE Industrial, and Organizational psychology | | | | | | | | | |
| configural (c)  metric (m)  scalar (s)  partial^e^ scalar (ps) | 49.34  59.00  83.36  61.31 | 4  7  10  8 | -  m-c  s-m  ps-m | -  5.77  24.15***  1.09 | -  2  2  1 | .966  .961  .945  .960 | -  -.005  -.016  -.001 | .143  .116  .115  .109 | -  -.027  -.001  -.007 |
| SE Clinical psychology | | | | | | | | | |
| configural (c)  metric (m)  partial^f^ metric  scalar (s)  partial^g^ scalar (ps) | 109.05  132.44  121.33  168.64  122.89 | 4  7  6  9  7 | -  m-c  pm-c  s-pm  ps-pm | -  10.38*  3.91  45.33***  0.06 | -  3  2  3  1 | .933  .920  .927  .899  .926 | -  -.013  .007  -.028  -.001 | .217  .179  .186  .179  .172 | -  -.038  -.031  -.007  -.014 |
| SE Educational psychology | | | | | | | | | |
| configural (c)  metric (m)  partial^h^ metric  scalar (s)  partial^i^ scalar (ps) | 72.34  90.62  81.85  114.52  82.33 | 4  7  6  9  7 | -  m-c  pm-c  s-pm  ps-pm | -  10.25*  .091  32.07***  1.68 | -  3  2  3  2 | .953  .942  .948  .927  .948 | -  -.011  -.005  -.021  .000 | .175  .146  .150  .145  .139 | -  -.029  -.025  -.005  -.011 |
| SE General | | | | | | | | | |
| configural (c)  metric (m)  scalar (s)  partial^j^ scalar (ps) | 293.07  132.77  162.77  137.60 | 4  7  10  8 | -  m-c  s-m  ps-m | -  1.33  23.26***  1.78 |  | .780  .904  .884  .901 | -  3  3  1 | .361  .180  .166  .171 | -  -.181  -.014  -.009 |

*Note.* SE = Self-efficacy; *χ*² = Chi-Square for all models is *p* < .001; *df* = degrees of freedom; CFI = comparative fit index; RMSEA = root mean square error of approximation; SB*χ2* = Satorra-Bentler scaled chi-square difference test; ΔCFI ≥ │.0.10│, ΔRMSEA ≥│.015│ signal lack of invariance between nested models; configural = factor loadings are invariant; metric = factor loadings and intercepts are invariant; scalar = factor loadings, intercepts, and residuals are invariant, partial scalar = factor loadings, and residuals are invariant, intercepts are partial invariant.

^a^Intercept I1 released.

^b^Intercept C3 released.

^c^Intercept E3, E4 released.

^d^Intercept G3, G4 released.

^e^Intercept I1, I3 released.

^f^Item C1 released.

^g^Intercept C1, C3 released.

^h^Item E2 released.

^i^Intercept E1, E3 released.

^j^Intercept G2, G4 released.

**p* < .05. ***p* < .01. ****p < .*001.

**Table A7. Standardized factor loadings of ability self-concepts/self-efficacy items**

**as obtained in Model A (*g*-factor model) and Model B (first-order correlated**

**factor model).**

| Item | IO-SC/ SE  (Model B) | Clin-SC/ SE  (Model B) | Edu-SC/ SE  (Model B) | GSC/GSE  (Model B) | g-SC/ SE  (Model A) |
| --- | --- | --- | --- | --- | --- |
| I1 | .89/ .78 |  |  |  | .81/ .65 |
| I2 | .90/ .80 |  |  |  | .75/ .67 |
| I3 | .96/ .70 |  |  |  | .80/ .61 |
| I4 | -/ .83 |  |  |  | -/ .73 |
| C1 |  | .92/ .81 |  |  | .88/. 74 |
| C2 |  | .91/ .81 |  |  | .83/ .74 |
| C3 |  | .96/ .72 |  |  | .89/ .67 |
| C4 |  | -/ .83 |  |  | -/ .77 |
| E1 |  |  | .92/ .80 |  | .87/ .73 |
| E2 |  |  | .91/ .80 |  | .83/ .73 |
| E3 |  |  | .94/ .72 |  | .87/ .68 |
| E4 |  |  | -/ .83 |  | -/ .78 |
| G1 |  |  |  | .89/ .79 | .71/ .66 |
| G2 |  |  |  | .91/ .79 | .62/ .68 |
| G3 |  |  |  | .97/ .71 | .70/ .61 |
| G4 |  |  |  | -/ .79 | -/ .71 |

*Note.* IO-SC/ SE = Ability self-concept/self-efficacy in Industrial and Organizational psychology; Clin-SC/SE

= Ability self-concept/self-efficacy in Clinical psychology; Edu-SC/ SE = Ability self-concept/self-efficacy in Educational psychology; g-SC/SE = General self-concept/self-efficacy in undergraduate psychology courses. All

All factor loadings were statistically significantly different from zero, *p* < .001.

**Table A8. Standardized factor loadings of ability self-concepts/self-efficacy items**

**as obtained in Model C (second-order correlated factor model).**

| Item | IO-SC/ SE | Clin-SC/ SE | Edu-SC/ SE | GSC/GSE | g-SC/ SE |
| --- | --- | --- | --- | --- | --- |
| I1 | .89/ .78 |  |  |  |  |
| I2 | .90/ .80 |  |  |  |  |
| I3 | .96/ .70 |  |  |  |  |
| I4 | -/ .83 |  |  |  |  |
| C1 |  | .92/ .81 |  |  |  |
| C2 |  | .91/ .81 |  |  |  |
| C3 |  | .96/ .72 |  |  |  |
| C4 |  | -/ .83 |  |  |  |
| E1 |  |  | .92/ .80 |  |  |
| E2 |  |  | .91/ .80 |  |  |
| E3 |  |  | .94/ .72 |  |  |
| E4 |  |  | -/ .83 |  |  |
| G1 |  |  |  | .89/ .79 |  |
| G2 |  |  |  | .81/ .79 |  |
| G3 |  |  |  | .97/ .71 |  |
| G4 |  |  |  | -/ .79 |  |
| IO-SC/SE |  |  |  |  | .79/ .83 |
| Clin-SC/ SE |  |  |  |  | .90/ .88 |
| Edu-SC/SE |  |  |  |  | .91/ .87 |
| GSC/GSE |  |  |  |  | .68/ .85 |

*Note.* IO-SC/ SE = Ability self-concept/self-efficacy in Industrial and Organizational psychology; Clin-SC/SE

= Ability self-concept/self-efficacy in Clinical psychology; Edu-SC/ SE = Ability self-concept/self-efficacy in Educational psychology; g-SC/SE = General self-concept/self-efficacy in undergraduate psychology courses. All

All factor loadings were statistically significantly different from zero, *p* < .001.

**Table A9. Standardized factor loadings of ability self-concepts/self-efficacy**

**items as obtained in Model D (incomplete bifactor model).**

| Item | IO-SC/ SE | Clin-SC/ SE | Edu-SC/ SE | Stat-SC/ SE | g-SC/ SE |
| --- | --- | --- | --- | --- | --- |
| I1 | .66/.55 |  |  |  | .59/.57 |
| I2 | 75/.47 |  |  |  | .49/.55 |
| I3  I4 | .79/.49  - /.58 |  |  |  | .56/.51  - /.58 |
| C1 |  | .70/.52 |  |  | .60/.64 |
| C2 |  | .75/.55 |  |  | .52/.58 |
| C3  C4 |  | .76/.47  - /.56 |  |  | .58/.55  - /.60 |
| E1 |  |  | .71/.57 |  | .58/.57 |
| E2 |  |  | .73/.58 |  | .54/.55 |
| E3  E4 |  |  | .77/.48  - /.57 |  | .56/.55  - /.59 |
| S1 |  |  |  | .58/.67 | .65/.47 |
| S2 |  |  |  | .69/.71 | .55/.54 |
| S3  S4 |  |  |  | .74/.60  - /.53 | .61/.61  - /.55 |
| G1 |  |  |  |  | .90/.81 |
| G2 |  |  |  |  | .80/.77 |
| G3  G4 |  |  |  |  | .97/.73  - /.77 |

*Note.* IO-SC/ SE = Ability self-concept/self-efficacy in Industrial and Organizational psychology; Clin-SC/SE

= Ability self-concept/self-efficacy in Clinical psychology; Edu-SC/ SE = Ability self-concept/self-efficacy in Educational psychology; Stat-SC/SE = Ability self-concept/self-efficacy in Statistics; g-SC/SE = General self-concept/self-efficacy in undergraduate psychology courses.

All factor loadings were statistically significantly different from zero, *p* < .001.

**Table A10. Correlations between loadings of ability self-concepts/self-efficacy factors within the combined first-order factor models with four first-order factors (Model B1).**

|  | IO | Clin | Edu | Gen |
| --- | --- | --- | --- | --- |
| IO | - |  |  |  |
| Clin | .693*** | - |  |  |
| Edu | .704*** | .839*** | - |  |
| Gen | - | - | - | - |

*Note*. SC = Ability self-concept; SE = Self-efficacy; IO = Industrial and organizational psychology; Clin = Clinical psychology; Edu = Educational psychology; Gen = General.

**p* < .05. ***p* < .01. ****p < .*001.

**Table A11. Correlations between ability self-concepts/self-efficacy factors within the combined first-order factor model with eight first-order factors (Model B2).**

|  | SC IO | SC Clin | SC Edu | SC Gen | SE IO | SE Clin | SE Edu |
| --- | --- | --- | --- | --- | --- | --- | --- |
| SC IO | - |  |  |  |  |  |  |
| SC Clin | .702*** | - |  |  |  |  |  |
| SC Edu | .717*** | .816*** | - |  |  |  |  |
| SC Gen | .580*** | .599*** | .597*** | - |  |  |  |
| SE IO | .782*** | .571*** | .591*** | .447*** | - |  |  |
| SE Clin | .596*** | .755*** | .616*** | .451*** | .704*** | - |  |
| SE Edu | .567*** | .630*** | .717*** | .466*** | .728*** | .766*** | - |
| SE Gen | .536*** | .565*** | .529*** | .602*** | .710*** | .748*** | .714*** |

*Note*. SC = Ability self-concept; SE = Self-efficacy; IO = Industrial and organizational psychology; Clin = Clinical psychology; Edu = Educational psychology; Gen = General.

**p* < .05. ***p* < .01. ****p < .*001.

**Table A12. Correlations between ability self-concepts/self-efficacy factors within the combined incomplete bifactor model with four first-order factors and one *g*-factor (Model D1).**

|  | IO | Clin | Edu | Gen |
| --- | --- | --- | --- | --- |
| IO | - |  |  |  |
| Clin | .506*** | - |  |  |
| Edu | .544*** | .680*** | - |  |
| Gen | - | - | - | - |

*Note*. SC = Ability self-concept; SE = Self-efficacy; IO = Industrial and organizational psychology; Clin = Clinical psychology; Edu = Educational psychology; Gen = General.

**p* < .05. ***p* < .01. ****p < .*001.

**Table A13. Standardized factor loadings of ability self-concepts/self-efficacy items**

**as obtained in the combined first-order factor model with four first-order factors (Model B1).**

| Item | IO (SC/ SE) | Clin (SC/ SE) | Edu (SC/SE) | GSC/GSE |
| --- | --- | --- | --- | --- |
| I1 | .88/ .60 |  |  |  |
| I2 | .90/ .61 |  |  |  |
| I3 | .94/ .61 |  |  |  |
| I4 | -/ .69 |  |  |  |
| C1 |  | .92/ .60 |  |  |
| C2 |  | .91/ .62 |  |  |
| C3 |  | .95/ .58 |  |  |
| C4 |  | -/ .64 |  |  |
| E1 |  |  | .91/ .58 |  |
| E2 |  |  | .91/ .61 |  |
| E3 |  |  | .93/ .57 |  |
| E4 |  |  | -/ .66 |  |
| G1 |  |  |  | .89/ .55 |
| G2 |  |  |  | .81/ .53 |
| G3 |  |  |  | .91/ .53 |
| G4 |  |  |  | -/ .58 |

*Note.* IO-SC/ SE = Ability self-concept/self-efficacy in Industrial and Organizational psychology; Clin-SC/SE = Ability self-concept/self-efficacy in Clinical psychology; Edu-SC/ SE = Ability self-concept/self-efficacy in Educational psychology; g-SC/SE = General self-concept/self-efficacy in undergraduate psychology courses. All All factor loadings were statistically significantly different from zero, *p* < .001.

**Table A14. Standardized factor loadings of ability self-concepts/self-efficacy items**

**as obtained in the combined first-order factor models with eight first-order factors (Model B2).**

| Item | IO-SC/ SE | Clin-SC/ SE | Edu-SC/SE | GSC/GSE |
| --- | --- | --- | --- | --- |
| I1 | .88/ .77 |  |  |  |
| I2 | .90/ .78 |  |  |  |
| I3 | .96/ .72 |  |  |  |
| I4 | -/ .84 |  |  |  |
| C1 |  | .92/ .80 |  |  |
| C2 |  | .91/ .80 |  |  |
| C3 |  | .96/ .72 |  |  |
| C4 |  | -/ .83 |  |  |
| E1 |  |  | .92/ .78 |  |
| E2 |  |  | .91/ .80 |  |
| E3 |  |  | .94/ .73 |  |
| E4 |  |  | -/ .85 |  |
| G1 |  |  |  | .90/ .78 |
| G2 |  |  |  | .81/ .78 |
| G3 |  |  |  | .96/ .72 |
| G4 |  |  |  | -/ .80 |

*Note.* IO-SC/ SE = Ability self-concept/self-efficacy in Industrial and Organizational psychology; Clin-SC/SE = Ability self-concept/self-efficacy in Clinical psychology; Edu-SC/ SE = Ability self-concept/self-efficacy in Educational psychology; g-SC/SE = General self-concept/self-efficacy in undergraduate psychology courses. All All factor loadings were statistically significantly different from zero, *p* < .001.

**Table A15. Standardized factor loadings of ability self-concepts/self-efficacy items**

**as obtained in the combined second-order factor models with eight first-order factors and one *g*-factor (Model C1)**

| Item | IO-SC/ SE | Clin-SC/ SE | Edu-SC/SE | GSC/GSE | g-(SC/SE) |
| --- | --- | --- | --- | --- | --- |
| I1 | .88/ .74 |  |  |  |  |
| I2 | .90/ .77 |  |  |  |  |
| I3 | .96/ .70 |  |  |  |  |
| I4 | -/ .82 |  |  |  |  |
| C1 |  | .92/ .77 |  |  |  |
| C2 |  | .92/ .79 |  |  |  |
| C3 |  | .96/ .70 |  |  |  |
| C4 |  | -/ .81 |  |  |  |
| E1 |  |  | .92/ .76 |  |  |
| E2 |  |  | .91/ .78 |  |  |
| E3 |  |  | .94/ .71 |  |  |
| E4 |  |  | -/ .81 |  |  |
| G1 |  |  |  | .90/ .75 |  |
| G2 |  |  |  | .81/ .74 |  |
| G3 |  |  |  | .96/ .80 |  |
| G4 |  |  |  | -/ .77 |  |
| IO-SC |  |  |  |  | .80 |
| Clin-SC |  |  |  |  | .87 |
| Edu-SC |  |  |  |  | .87 |
| G-SC |  |  |  |  | .66 |
| IO-SE |  |  |  |  | .81 |
| Clin-SE |  |  |  |  | .85 |
| Edu-SE |  |  |  |  | .84 |
| G-SE |  |  |  |  | .78 |

*Note.* IO-SC/ SE = Ability self-concept/self-efficacy in Industrial and Organizational psychology; Clin-

SC/SE= Ability self-concept/self-efficacy in Clinical psychology; Edu-SC/ SE = Ability self-concept/self-efficacy in Educational psychology; g-SC/SE = General self-concept/self-efficacy in undergraduate psychology courses. AllAll factor loadings were statistically significantly different from zero, p < .001.

**Table A16. Standardized factor loadings of ability self-concepts/self-efficacy items**

**as obtained in the combined second-order factor models with eight first-order factors**

**and two *g*-factors (Model C2).**

| Item | IO-SC/ SE | Clin-SC/ SE | Edu-SC/SE | GSC/GSE | g-SC/SE |
| --- | --- | --- | --- | --- | --- |
| I1 | .89/ .77 |  |  |  |  |
| I2 | .90/ .79 |  |  |  |  |
| I3 | .96/ .71 |  |  |  |  |
| I4 | -/ .84 |  |  |  |  |
| C1 |  | .92/ .90 |  |  |  |
| C2 |  | .91/ .81 |  |  |  |
| C3 |  | .96/ .72 |  |  |  |
| C4 |  | -/ .84 |  |  |  |
| E1 |  |  | .92/ .79 |  |  |
| E2 |  |  | .91/ .80 |  |  |
| E3 |  |  | .94/ .72 |  |  |
| E4 |  |  | -/ .85 |  |  |
| G1 |  |  |  | .90/ .78 |  |
| G2 |  |  |  | .81/ .78 |  |
| G3 |  |  |  | .97/ .72 |  |
| G4 |  |  |  | -/ .80 |  |
| IO-SC |  |  |  |  | .80 |
| Clin-SC |  |  |  |  | .90 |
| Edu-SC |  |  |  |  | .90 |
| G-SC |  |  |  |  | .68 |
| IO-SE |  |  |  |  | .83 |
| Clin-SE |  |  |  |  | .89 |
| Edu-SE |  |  |  |  | .87 |
| G-SE |  |  |  |  | .83 |

*Note.* IO-SC/ SE = Ability self-concept/self-efficacy in Industrial and Organizational psychology; Clin-SC/SE = Ability self-concept/self-efficacy in Clinical psychology; Edu-SC/ SE = Ability self-concept/self-efficacy in Educational psychology; g-SC/SE = General self-concept/self-efficacy in undergraduate psychology courses. All All factor loadings were statistically significantly different from zero, *p* < .001.

**Table A17. Standardized factor loadings of ability self-concepts/self-efficacy**

**items as obtained in the incomplete bifactor model with four first-order factors**

**and one *g*-factor (Model D1).**

| Item | IO(SC/ SE) | Clin (SC/ SE) | Edu (SC/ SE) | | G(SC/SE) | | g-(SC/SE) |
| --- | --- | --- | --- | --- | --- | --- | --- |
| I1 | .71/ .41 |  |  |  | | .49/ .50 | |
| I2 | .77/ .46 |  |  |  | | .56/ .38 | |
| I3 | .79/ .48 |  |  |  | | .52/ .35 | |
| I4 | / .54 |  |  |  | | .-/ .42 | |
| C1 |  | .69/ .41 |  |  | | .60/ .49 | |
| C2 |  | .73/ .46 |  |  | | .53/. 42 | |
| C3 |  | .73/ .44 |  |  | | .60/ .37 | |
| C4 |  | -/ .47 |  |  | | -/ .43 | |
| E1 |  |  | .69/ .40 |  | | .58/ .47 | |
| E2 |  |  | .73/ .44 |  | | .55/ .41 | |
| E3 |  |  | .74/ .40 |  | | .57/ .40 | |
| E4 |  |  | -/ .46 |  | | -/ .47 | |
| G1 |  |  |  |  | | .89/ .63 | |
| G2 |  |  |  |  | | .81/ .56 | |
| G3 |  |  |  |  | | .91/ .55 | |
| G4 |  |  |  |  | | -/ .60 | |

*Note.* IO-SC/ SE = Ability self-concept/self-efficacy in Industrial and Organizational psychology; Clin-SC/SE

= Ability self-concept/self-efficacy in Clinical psychology; Edu-SC/ SE = Ability self-concept/self-efficacy in Educational psychology; Stat-SC/SE = Ability self-concept/self-efficacy in Statistics; g-SC/SE = General self-concept/self-efficacy in undergraduate psychology courses.

All factor loadings were statistically significantly different from zero, *p* < .001.
